# Deregulation of apoptosis-related genes is associated with *PRV1 *overexpression and JAK2 V617F allele burden in Essential Thrombocythemia and Myelofibrosis

**DOI:** 10.1186/1756-8722-5-2

**Published:** 2012-02-02

**Authors:** Raquel Tognon, Elainy PL Gasparotto, Renata P Neves, Natália S Nunes, Aline F Ferreira, Patrícia VB Palma, Simone Kashima, Dimas T Covas, Mary Santana, Elizabeth X Souto, Maria Aparecida Zanichelli, Belinda P Simões, Ana Maria de Souza, Fabíola A Castro

**Affiliations:** 1 Department of Clinical, Toxicological and Bromatological Analysis, University of São Paulo, Ribeirão Preto School of Pharmaceutical Sciences, Ribeirão Preto, Brazil; 2 Regional Blood Center of Ribeirão Preto, Clinical Hospital, University of São Paulo, Ribeirão Preto School of Medicine, Ribeirão Preto, Brazil; 3 Brigadeiro Hospital of São Paulo, São Paulo, Brazil; 4 Institute for Cancer Treatment in Children-ITACI, São Paulo, Brazil; 5 Department of Clinical Medicine, University of São Paulo, Ribeirão Preto School of Medicine, Ribeirão Preto, Brazil; 6 INCT-IF-CNPq

**Keywords:** Chronic Myeloproliferative Neoplasms, Apoptosis, JAK2 V617F allele burden, *PRV1 *, BCL2 family members

## Abstract

**Background:**

Essential Thrombocythemia (ET) and Primary Myelofibrosis (PMF) are Chronic Myeloproliferative Neoplasms (MPN) characterized by clonal myeloproliferation/myeloaccumulation without cell maturation impairment. The JAK2 V617F mutation and *PRV1 *gene overexpression may contribute to MPN physiopathology. We hypothesized that deregulation of the apoptotic machinery may also play a role in the pathogenesis of ET and PMF. In this study we evaluated the apoptosis-related gene and protein expression of BCL2 family members in bone marrow CD34^+ ^hematopoietic stem cells (HSC) and peripheral blood leukocytes from ET and PMF patients. We also tested whether the gene expression results were correlated with JAK2 V617F allele burden percentage, *PRV1 *overexpression, and clinical and laboratory parameters.

**Results:**

By real time PCR assay, we observed that *A1, MCL1, BIK and BID*, as well as *A1, BCLW *and *BAK *gene expression were increased in ET and PMF CD34^+ ^cells respectively, while pro-apoptotic *BAX *and anti-apoptotic *BCL2 *mRNA levels were found to be lower in ET and PMF CD34^+ ^cells respectively, in relation to controls. In patients' leukocytes, we detected an upregulation of anti-apoptotic genes *A1, BCL2, BCL-X_L _*and *BCLW*. In contrast, pro-apoptotic *BID *and *BIM_EL _*expression were downregulated in ET leukocytes. Increased BCL-X_L _protein expression in PMF leukocytes and decreased BID protein expression in ET leukocytes were observed by Western Blot. In ET leukocytes, we found a correlation between JAK2 V617F allele burden and *BAX, BIK and BAD *gene expression and between *A1, BAX *and *BIK *and *PRV1 *gene expression. A negative correlation between *PRV1 *gene expression and platelet count was observed, as well as a positive correlation between *PRV1 *gene expression and splenomegaly.

**Conclusions:**

Our results suggest the participation of intrinsic apoptosis pathway in the MPN physiopathology. In addition, *PRV1 *and JAK2 V617F allele burden were linked to deregulation of the apoptotic machinery.

## Background

Essential Thrombocythemia (ET) and Primary Myelofibrosis (PMF) are disorders classified as Philadelphia chromosome-negative Myeloproliferative Neoplasms (MPN) [1]. ET is a clonal disease characterized by an increase in the platelet count associated with bone marrow megakaryocyte hyperplasia. Thrombosis and hemorrhagic events are the main co-morbities in ET patients. PMF is characterized by bone marrow fibrosis, as well as peripheral blood findings such as anemia, leukoerythroblastosis and the presence of dacryocytes in peripheral blood [2].

The JAK2 V617F mutation, which leads to constitutive JAK2 activation, was shown to play an important role in MPN pathogenesis, and is found in 95% of Polycythemia Vera (PV) patients and in at least 50% of ET and PMF patients [3]. Constitutive JAK2 activation triggers several signaling pathways linked to cell survival and proliferation promoting myeloproliferation and resistance to cell death [4-8]. Other mutations have recently been described in ET and PMF patients, such as mutations in *JAK2 *exon 12 and in the *TET2, CBL, MPL *and *AXSL *genes [9-13]. Several studies suggest an association between MPN clinical features and the JAK2 V617F allele burden [14]. Although this knowledge and the identification of these additional mutations greatly enhanced our understanding of MPN physiopathology, a complete understanding of the cellular and molecular mechanisms involved is still lacking.

Another relevant molecular alteration described in MPN patients is the overexpression of *PRV1*, a surface receptor from hematopoietic cells associated with cell proliferation [15] related to JAK/STAT and SRC kinase pathways [16]. *PRV1 *overexpression was initially described in PV patients and in some cases of ET, but was not found elevated in other malignant hematological diseases such as Chronic Myeloid Leukemia (CML) [15,17,18]. Considering that *PRV1 *gene expression is deregulated in MPN, it has been suggested that it may be used as a molecular marker in the diagnosis of these diseases [19]. Literature also supports the hypothesis that *PRV1 *overexpression contributes to MPN physiopathology, considering that there are studies showing a correlation between *PRV1 *expression and patients' clinical and laboratory features [15].

The apoptosis process may be triggered by two major pathways: the extrinsic or death-receptors pathway, and the intrinsic or mitochondrial pathway. The intrinsic apoptosis pathway or mitochondrial pathway is triggered by several stimuli such as ultra violet radiation, growth factor/cytokine deprivation, chemotherapeutic agents, viral infection and other stress factors related to physical and chemical injuries [20]. This pathway is mainly regulated by BCL2 family members, classified as either anti-apoptotic (BCL2, BCL-X_L_, BCLW, MCL1 and A1) or pro-apoptotic (BAX, BAK, BAD, BID, BIM, Bok, BIK, BMF, BOO, BCL-X_S_, PUMA and NOXA) proteins [21]. The apoptotic process was found to be deregulated in several hematopoietic neoplasms, leading to resistance to therapy and progression of disease. Alterations in apoptosis have been described in CML, Myelodysplastic Syndrome (MDS), Acute Myeloid Leukemia (AML), PV, ET and PMF [20,22-24].

In this study we investigated the potential association between apoptosis deregulation, JAK2 mutation and *PRV1 *overexpression in ET and PMF patients. We focused on evaluating the expression of apoptosis-related genes of the BCL2 family, JAK2 V617F allele burden and *PRV1 *expression in ET and PMF in bone marrow CD34^+ ^hematopoietic stem cells (HSC) and peripheral blood leukocytes from ET and PMF patients. The correlation between gene expression, JAK2 allele burden, clinical and laboratory parameters and *PRV1 *expression were also assessed.

We observed a deregulation in the expression of most of the studied genes in bone marrow CD34^+ ^cells and peripheral leukocytes from ET and PMF patients in comparison to controls. Furthermore, a correlation between *BAD, BAX *and *BIK *expression and JAK2 V617F allele burden as well as between *A1, BAX *and *BIK *and *PRV1 *expression was detected. A negative correlation between *PRV1 *expression and platelet count was also observed, as well as a positive correlation between *PRV1 *expression and splenomegaly.

## Subjects and methods

### Patients and Controls

We studied bone marrow CD34^+ ^HSC and peripheral blood leukocytes from 26 ET patients (5 male, 21 female, mean age: 60.2 years; range: 35-80y) and 12 PMF patients (9 male, 3 female, mean age: 61.7 years, range: 41-80y), without treatment. The bone marrow control group was composed of 23 bone marrow donors from the Bone Marrow Transplantation Unit of the Clinical Hospital - University of São Paulo at the Ribeirão Preto School of Medicine, São Paulo, Brazil. This group included 12 male and 11 female, with a mean age of 35.9 years (range 14 -54y). We also collected peripheral blood samples from 37 individuals with similar age, skin color and gender (14 male, 23 female with mean age of 58.5 years, range 31-80y) for leukocyte isolation. This research was approved by the local ethics committees and the consent form was signed by the patients and volunteer controls. It was not possible to match the age and sex of bone marrow donors because the local ethics committees only permit bone marrow samples to be obtained from bone marrow donors during the bone marrow transplantation cell collection procedure.

### JAK2 V617F allele burden detection, platelet count and spleen size determination

Detection of the JAK2 V617F mutation and the allele burden were determined as described in Tognon et al., 2011 [24], by real time allelic discrimination PCR assay. Platelets count from ET and PMF peripheral blood was determined by the Abbott Cell Dyn 3500SL Hematology Analyzer. Spleen size was determined by ultrasonography. In order to correlate gene expression and splenomegaly, we used the increase in centimeters (cm) along the longer dimension of the patients' spleen, considering as reference, a value of 12 cm.

### Cell Isolation, RNA extraction and cDNA synthesis

Cell isolation, RNA extraction and cDNA synthesis were performed as described in Tognon et al., 2011 [24]. Briefly, bone marrow CD34^+ ^HSC were separated using the Ficoll-Hypaque protocol followed by Miltenyi CD34 isolation kit MidiMacs CD34^+ ^Isolation Kit (MACS; Miltenyi Biotec, Bergisch Gladbach, Germany) and peripheral leukocytes were obtained by the Haes-Steril method. Total RNA from CD34^+ ^HSC and leukocytes was extracted according to the Trizol™ (Invitrogen Life Technologies, CarlsBAD, California, USA) method. One microgram of RNA was used to synthesize cDNA using the High Capacity™ Kit from Applied Biosystems Life Technologies (Foster City, California, USA).

### Quantification of apoptosis-related gene and PRV1 expression

The expression of anti-apoptotic genes *A1, MCL1, BCL2, BCL-X_L _*and *BCLW *and pro-apoptotic genes *BAD, BAX, BAK, BID, BIK *and *BIM_EL _*was evaluated by real time PCR in duplicate. For gene expression quantification we used the SYBR Green PCR Master Mix Kit (Applied Biosystems) and specific oligonucleotides (Invitrogen Life Technologies) (Table 1) on the 7500 Real Time PCR System (Applied Biosystems Life Technology). The results were normalized by the geometric mean of the *beta-actin *and the *GAPDH *housekeeping genes expression and represented by 2^-ΔΔCt ^as described by Tognon et al., 2011 [24].

**Table 1:** Real time PCR primer sequences

*Gene*	Primer Sequence
*A1*	F: GGC TGG CTC AGG ACT ATC R: CCA GTT AAT GAT GCC GTC
*MCL1*	F: AGA AAG CTG CAT CGA ACC AT R: CC AGC TCC TAC TCC AGC AAC
*BCL2*	F: ACG AGT GGG ATG CGG GAG ATG TG R: GCG GTA GCG GCG GGA GAA GTC
*BCLW*	F: AGT TCG AGA CCC GCT TCC R: CCC GTC CCC GTA TAG AGC
*BCL-X_L _*	F: CTG AAT CGG AGA TGG AGA CC R: TGG GAT GTC AGG TCA CTG AA
*BID*	F: GCT TCC AGT GTA GAC GGA GC R: GTG CAG ATT CAT GTG TGG ATG
*BIK*	F: TCT GCA ATT GTC ACC GGT TA R: TTG AGC ACA CCT GCT CCT C
*BIM_EL _*	F: GCC CCT ACC TCC CTA CAG AC R: AAG ATG AAA AGC GGG GAT CT
*BAD*	F: CCG AGT GAG CAG GAA GAC TC R: GGT AGG AGC TGT GGC GAC T
*BAK*	F: TCT GGC CCT ACA CGT CTA CC R: ACA AAC TGG CCC AAC AGA AC
*BAX*	F: CCC TTT TGC TTC AGG GTT TC R: TCT TCT TCC AGA TGG TGA GTG
*β-actin*	F: GCC CTG AGG CAC TCT TCC A R: CCA GGG CAG TGA TCT CCT TCT
*GAPDH*	F: GCCTCAAGATCATCAGCAATGC R: CATGGACTGTGGTCATGAGTCCT

F: Forward Primer, R: Reverse Primer

The *PRV1 *gene was quantified by the TaqMan PCR Master Mix kit using TaqMan^® ^Gene Expression Assay (*PRV1 *- Hs00360669_m1; *GAPDH *-Hs99999905_m1) on Mastercycler^® ^ep Realplex (Eppendorf AG, Hamburg, Germany). For this gene, *GAPDH *was used as housekeeping gene.

### Western Blot

One million leukocytes were lysated in 100 uL of sample buffer (5% mercaptoethanol, 4% sodium dodecyl sulfate - SDS, 20% glycerol and 100 mM Tris-HCl-pH 6.8), boiled at 100°C for 5 minutes and kept at -20°C until the *Western-Blot *analysis was performed. Twenty-five ml of patient and controls' lysate were loaded in polyacrilamide gel, the proteins were separated on 15% SDS-PAGE and transferred onto polyvinylidene difluoride (PVDF) membrane (Amersham, GE Healthcare Life Science). The membranes were incubated with the primary antibody anti-BCL-X_L _(1:200 dilution, H-62, SC7195, Santa Cruz Biotechnology^®^) or anti-BID (1:500 dilution, # 611528, BD Pharmingen) diluted in TBS-Tween (20 mM Tris, 137 mM NaCl, 0.01% Tween-20) with 5% non-fat milk for 16 hours. As a control for sample loading, the blot was probed with anti-γ-tubulin (1:2000 dilution, T3320, Sigma-Aldrich). Horseradish peroxidase (HRP) conjugated secondary antibodies and ECL Plus^® ^(Amersham, GE Healthcare Life Science) were used for protein expression detection. Protein expression was measured by densitometry analysis performed by ImageQuant TL (Image Analysis Software, 2005, Amersham). To express the proteins densitometry results, firstly we calculated the ratio between protein test IDV (Integrated density value) and tubulin IDV, and then we calculated the protein expression ratio between patient and controls (IDV of patients protein expression divided by controls protein expression).

### Statistical analysis

The Mann-Whitney test (t-test, one-tailed) was used to compare the values of gene expression among the studied groups. The correlations between apoptosis-related gene expression, JAK2 allele burden and *PRV1 *gene expression were carried out by Spearman tests using Prisma 5.0 Software. A *p *value < 0.05 was taken as significant.

## Results

### Gene expression of apoptosis-related BCL2 family members is deregulated in ET and PMF patients

**Figure 1:**
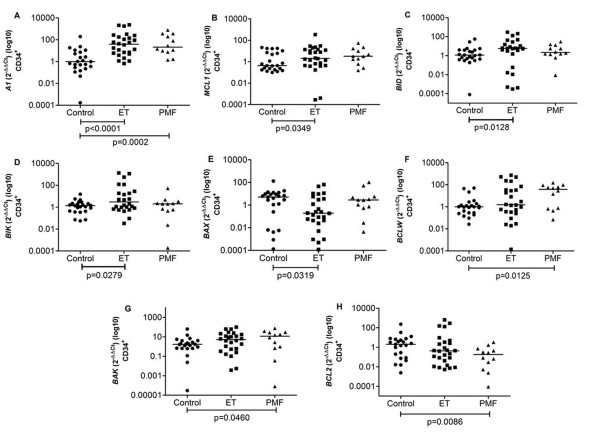
**Gene expression in Control, ET and PMF CD34^+ ^HSC**. (A) anti-apoptotic gene *A1 *was increased in ET and PMF compared to control; (B) anti-apoptotic gene *MCL1 *was increased in ET compared to control; (C) pro-apoptotic gene *BID *was increased in ET compared to control; (D) pro-apoptotic gene *BIK *was increased in ET compared to control; (E) pro-apoptotic gene *BAX *was decreased in ET compared to control; (F) anti-apoptotic gene *BCLW *was increased in PMF compared to control; (G) pro-apoptotic gene BAK was increased in ET compared to control; (H) anti-apoptotic gene *BCL2 *was decreased in PMF compared to control. The significant "p values" are shown in the figure. Otherwise, p > 0.05. The horizontal bars show the median 2^-ΔΔCt ^for each group.

Gene expression analysis in CD34^+ ^HSC from ET patients showed an increased expression of anti-apoptotic genes *A1 *and *MCL1 *and of pro-apoptotic genes *BID *and *BIK *(median = 39.25, 2.07, 5.69 and 2.99, respectively) in comparison to controls (0.986, 0.431, 1.15 and 1.36, respectively) (p < 0.0001, p = 0.0349, p = 0.0128 and p = 0.0279, respectively) (Figure 1A-D). The mRNA level of the pro-apoptotic gene *BAX *was found to be lower (0.19) in patients than controls (5.08) (p = 0.0319) (Figure 1E).

In PMF CD34^+ ^HSC the anti-apoptotic genes *A1, BCLW *and pro-apoptotic *BAK *expression were significantly increased (21.11, 1.55 and 11.67, respectively) compared to controls (0.99, 1.01 and 1.67, respectively) (p = 0.0002, p = 0.0125 and p = 0.0460, respectively) (Figure 1A, F and 1G). *BCL2 *mRNA levels were downregulated in these cells (0.18) in comparison to controls (1.99) (p = 0.0086) (Figure 1H).

**Figure 2:**
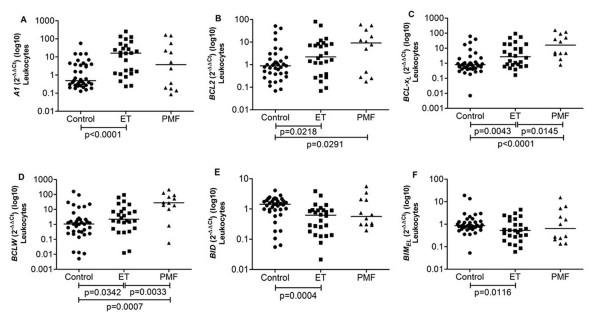
**Gene expression in Control, ET and PMF leukocytes**. (A) anti-apoptotic gene *A1 *was increased in ET compared to control; (B) anti-apoptotic gene *BCL2 *was increased in ET and PMF compared to control; (C) anti-apoptotic gene *BCL-X_L _*was increased in ET and PMF compared to control and also significantly different between ET and PMF; (D) anti-apoptotic gene *BCLW *was increased in ET and PMF compared to control and also significantly different between ET and PMF; (E) pro-apoptotic gene *BID *was decreased in ET compared to control; (F) pro-apoptotic gene *BIM_EL _*was decreased in ET compared to control. The significant "p values" are shown in the figure. Otherwise, p > 0.05. The horizontal bars show the median 2^-ΔΔCt ^for each group.

In ET patients' leukocytes we found an overexpression of the anti-apoptotic genes *A1, BCL2, BCL-X_L _*and *BCLW *(16.10, 2.21, 2.71 and 2.21, respectively) when compared with controls (0.49, 0.88, 0.80 and 1.06, respectively) (p < 0.0001, p = 0.0218, p = 0.0043 and p = 0.0342, respectively) (Figure 2A-D). The expression of pro-apoptotic genes *BID *and *BIM_EL _*was lower in ET leukocytes (0.62, 0.53) than in controls (1.43, 0.86) (p = 0.0004, p = 0.0116) (Figure 2E, F).

In PMF leukocytes, *BCL2, BCL-X_L _and BCLW *expression were elevated (9.17, 16.10, 27.86, respectively) compared to controls (0.88, 0.80 and 1.07, respectively) (p = 0.0291, p < 0.0001 and p = 0.0007, respectively) (Figure 2B, C and 2D).

Pro-apoptotic *BAD *expression was found to be increased in ET and PMF leukocytes (3.92, 5.18) compared to controls (0.63) (p = 0.0130, p = 0.0276) (data not shown).

There were no differences when we compared *BCL-X_L _, BAD *and *BIM_EL _*expression between CD34^+ ^cells from ET and PMF patients and controls, and this was also the case for *MCL1, BID, BIK *and *BAX *expression in leukocytes (*p *> 0.05). Furthermore, between ET and PMF leukocytes we only found a significant difference in *BCLW *and *BCL-X_L _*expression (p = 0.0145, p = 0.0033) (Figure 2C and 2D).

### BCL-X_L _*and *BID *protein levels were different between controls and PMF and ET patients*

**Figure 3:**
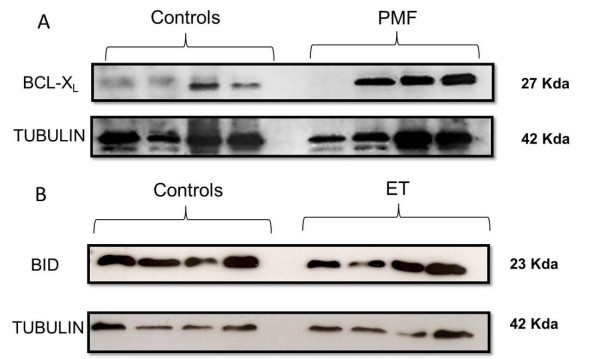
**Protein expression in Control, ET and PMF leukocytes**. (A) anti-apoptotic BCL-X_L _showed higher expression in PMF leukocyte than in controls (fold change: 2.4); (B) pro-apoptotic BID showed lower expression in ET leukocyte than in controls (fold change: 0.64). Tubulin was probed as loading control and densitometry was used to confirm the fold change in the protein expression.

We detected an elevated level of anti-apoptotic BCL-X_L _in PMF leukocytes and a decreased expression of pro-apoptotic BID protein in ET leukocytes in comparison to control subjects (Figure 3A and 3B). Densitometry quantification by Integrated Density Value (IDV) showed that BCL-X_L _protein level is 2.4 times higher in PMF leukocytes than in controls, and BID protein in ET leukocytes is 0.64 fold decreased in relation to its expression in controls.

### JAK2 V617F allele burden is correlated with BAD, BAX and BIK expression in ET patients' leukocytes

**Figure 4:**
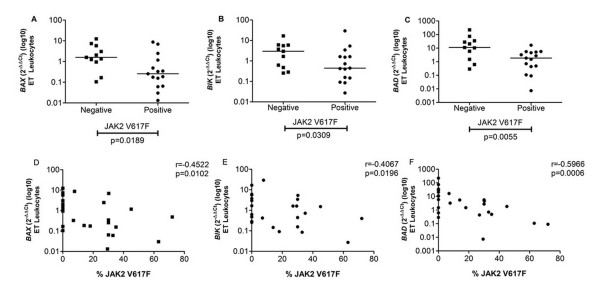
**Apoptosis-related gene expression versus JAK2 V617F mutation**. (A, B and C) pro-apoptotic genes *BAX, BIK *and *BAD *showed lower expression in ET JAK2 V617F positive patients compared to negative patients; (D, E and F) *BAX, BIK *and *BAD *expression in ET leukocytes also negatively correlated with the patients' JAK2 V617F allele burden. The significant "p values" and the "r value" for correlations are shown in the figure. The horizontal bars show the median 2^-ΔΔCt ^for each group.

In JAK2 V617F-positive ET patients we detected lower levels of pro-apoptotic *BAX, BIK *and *BAD *expression (median: 0.26, 0.45 and 1.86, respectively) in leukocytes compared to JAK2 V617F-negative ET patients (1.59, 2.96 and 11.10, respectively) (p = 0.0189, p = 0.0309 and p = 0.0055, respectively) (Figure 4A-C). In addition, *BAX, BIK *and *BAD *expression were negatively correlated with JAK2 V617F allele burden (*BAX*: r = -0.4522; p = 0.0102; *BIK*: r = -0.4067, p = 0.0196; *BAD*: r = -0.5966, p = 0.0006) in ET (Figure 4D-F).

### PRV1 overexpression is correlated with A1, BIK and BAX gene expression, JAK2 V617F mutation, platelet count and splenomegaly

**Figure 5:**
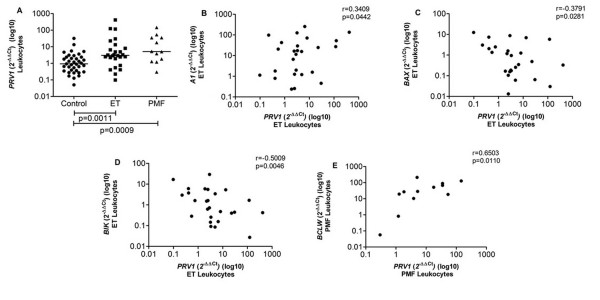
***PRV1 *gene expression in Control, ET and PMF leukocytes and *PRV1 *expression versus gene expression**. (A) *PRV1 *expression was significantly elevated in ET and PMF leukocytes; (B) anti-apoptotic gene *A1 *showed positive correlation with *PRV1 *expression in ET leukocytes; (C and D) pro-apoptotic genes *BAX *and *BIK *showed negative correlation with *PRV1 *expression in ET leukocytes; (E) anti-apoptotic gene *BCLW *showed positive correlation with *PRV1 *expression in PMF leukocytes. The significant "p values" are shown in the figure. Otherwise, p > 0.05. The horizontal bars show the median 2^-ΔΔCt ^for each group.

We observed that *PRV1 *was overexpressed in ET (3.04) and PMF (5.12) leukocytes in comparison to controls (0.93) (p = 0.0011 and p = 0.0009) (Figure 5A). In ET leukocytes we also found that *PRV1 *expression was positively correlated with *A1 *expression (r = 0.3409, p = 0.0442), and negatively correlated with expression of *BAX *(r=-0.3791, p = 0.0281) and *BIK *(r=-0.5009, p = 0.0046) (Figure 5B-D). Moreover, *PRV1 *expression was positively correlated with *BCLW *(r = 0.6503, p = 0.0110) expression in PMF patients' leukocytes (Figure 5E).

**Figure 6:**
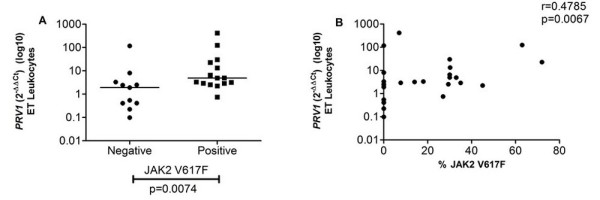
***PRV1 *expression versus JAK2 V617F mutation**. (A) *PRV1 *expression was higher in ET JAK2 V617F positive patients than in JAK2 V617F negative patients; (B) a positive correlation between *PRV1 *expression in ET leukocytes and JAK2 V617F allele burden percentage was detected. The significant "p values" and the "r value" for correlations are shown in the figure.

We detected a positive correlation between *PRV1 *expression and JAK2 V617F mutation allele burden in ET and PMF patients and this result corroborates the literature concerning the association between *PRV1 *and JAK2 V617F described in murine myeloid cells [25] and PV patients [26]. Leukocytes from ET patients harboring the JAK2 V617F mutation showed higher expression of *PRV1 *(median = 4.88) in comparison to those negative for the JAK2 V617F mutation (1.91) (p = 0.0074) (Figure 6A) and, consequently, a positive correlation between *PRV1 *expression and JAK2 V617F allele burden was observed in ET patient leukocytes (r = 0.4785; p = 0.0067) (Figure 6B).

**Figure 7:**
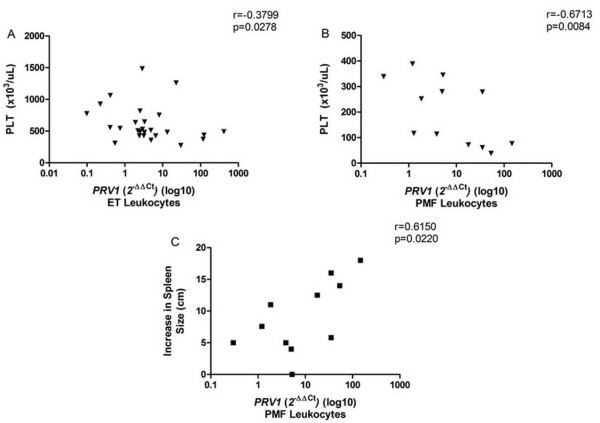
**Correlation of *PRV1 *gene expression with platelet count (PLT) and splenomegaly**. *PRV1 *expression in ET (A) and PMF (B) leukocytes showed negative correlation with platelets (PLT); (C) *PRV1 *expression in PMF leukocytes showed positive correlation with the increase in centimeters of the spleen length (splenomegaly) determined by ultrasonography (reference value: 12 cm). The significant "p values" and the "r" value for correlations are shown in the figure.

Furthermore, *PRV1 *expression was negatively correlated with platelet count in ET (r=-0.3799, p = 0.0278) (Figure 7A) and PMF patients (r=-0.6713, p = 0.0084) (Figure 7B), and *PRV1 *expression in PMF leukocytes showed a positive correlation with the increase of the spleen size in centimeters (splenomegaly) (r = 0.6150, p = 0.0220) (Figure 7C).

## Discussion

Our results indicate a deregulated expression of genes related to the intrinsic apoptosis pathway in CD34^+ ^HSC and peripheral leukocytes from ET and PMF patients.

Our hypothesis was that higher expression levels of anti-apoptotic genes may contribute to the myeloaccumulation in ET and PMF. In support of this notion, in this study we found increased expression of *A1, MCL1, BCLW *and *BCL-X_L _*genes in ET and PMF patients compared to controls. Importantly, as previously described by our group, the cells from MPN patients are resistant to apoptosis induced by different drugs (actinomycin D, teniposide, etoposide, cytarabin, and staurosporin) [24]. These observations, and our findings in the present investigation, support our hypothesis that deregulated expression of apoptosis-related genes is linked to myeloaccumulation and pathogenesis in ET and PMF.

The BCL2 family proteins have a central role in the process of apoptosis control. The A1, BCL2, BCLW, BCL-X_L _and MCL1 BCL2-family members encode anti-apoptotic molecules, while BAD, BAX, BAK, BID, BIK, BOK, BOO, PUMA, NOXA and BIM_EL _encode pro-apoptotic molecules, all of them involved in the mitochondrial apoptosis pathway [21].

*A1 *(*bfl1*) expression is detected in several tissues such as hematopoietic and endothelial cells [27]. *A1 *gene transcription is dependent on the NF-kB pathway and its overexpression has been reported in Chronic Lymphocytic Leukemia (CLL), particularly in CLL patients who do not respond to therapy [27].

*BCL2 *is an oncogene, which was first identified in Non-Hodgkin lymphoma B-cells and this gene is a pivotal molecule in the mitochondrial apoptosis pathway. Castro et al. (2005) described in the American Society Hematology Congress [28] a decrease in *BCL2 *expression and an increase in *BCL-X_L_, BCLW, A1, MCL1 *and *cflip *expression in CML and they also demonstrated that this profile of expression was correlated with CML progression. Furthermore, many publications have shown that in neoplasms such as breast or stomach cancer, high levels of BCL2 proteins were associated with a worse prognosis [29].

Deregulation of *MCL1*expression was described in hepatocellular carcinoma [30] and in multiple myeloma [31]. Furthermore, the expression of this gene was also found to be correlated with prognosis in multiple myeloma [31]. Such deregulated expression was also verified in bone marrow blasts from patients with MDS [22]. Del Poeta et al. reported increased levels of *BCL2, BCL-X_L _*and *MCL1 *expression in AML [23] and Aichberger et al. showed that *MCL1 *is a BCR-ABL target gene in CML [32].

Among the pro-apoptotic proteins of the BCL2 family, *BAX *has a crucial role in apoptosis and the lack of *BAX *leads to apoptosis impairment and facilitates the development of B-cell lymphoma by c-Myc stimulation [33].

Regarding MPN pathophysiology, Zhang et al. [34] demonstrated that BCL-X_L _is down-regulated early during *in vitro *differentiation of megakaryocytes from ET patients and this might reflect an early entry of megakaryocytes into a degenerating mature stage [34]. There is little data in the literature regarding apoptosis deregulation in PMF. On one hand, Mesa et al. (2006) showed that the levels of the anti-apoptotic and pro-apoptotic *BAX, BAK, BIM *and *Bmf *were not different between PMF and controls [35]. On the other hand, it was demonstrated that JAK2 inhibition in a cellular model of MPN (JAK2 V617F positive cell lineage) triggers BIM activation and leads to enhanced sequestration of MCL1, furthermore, BCL-X_L _and MCL1 depletion by RNAi was sufficient to compromise JAK2 V617F mutant cell viability and sensitized the cells to JAK2 inhibition, indicating an association between these apoptosis-related molecules and the aberrant JAK2 signaling in these cells [36].

It has been described that BCL2 family proteins interact with each other to control the intrinsic apoptosis pathway [37]. The balance among the activities of these proteins is very important to tightly control the apoptosis process [38]. The MPN patients enrolled in this study showed overexpression of several anti-apoptotic genes such as *A1 *and *MCL1 *but also overexpression of some pro-apoptotic genes such as *BIK, BID *and *BAK *in CD34^+ ^cells. Furthermore, in CD34^+ ^cells we observed a downregulation of *BAX *and *BID*, and *BIM_EL _*levels were found reduced in ET leukocytes. Therefore, we postulate that the balance among these BCL2 family members is disrupted, and may contribute to the myeloaccumulation in these patients due to the increase in cell survival.

In addition, we found that mRNA levels of pro-apoptotic *BAX, BAD *and *BIK *mRNAs were lower in JAK2 V617F-positive patients than in those negative, and also presented a negative correlation with the JAK2 V617F allele burden. Some reviews published in the literature have already pointed out and discussed the relationship between constitutively activated JAK2/STAT signaling and deregulation of apoptosis-related genes in CML and human tumor cell lines [39,40].

Moreover, we analyzed *PRV1 *mRNA expression and the correlation with JAK2 V617F mutation, and with clinical laboratorial data in leukocytes from control, ET and PMF patients. *PRV1 *is a hematopoietic cell surface receptor that has been shown to transduce intracellular signals leading to proliferation, involving JAK2 and Src kinases [16]. *PRV1 *is overexpressed in MPN patients [15,19,41,42] and seems to be associated with PV disease phenotype characterized by high erythrocyte and low platelet counts [15]. These studies also described a correlation between *PRV1 *expression and the JAK2 V617F allele burden, as well as between *PRV1 *overexpression and elevated JAK2 tyrosine kinase activity [15,19,25,41,42].

In our results we detected a correlation between *PRV1 *overexpression and the anti-apoptotic genes *A1 *and *BCLW*, and the pro-apoptotic genes *BAX *and *BIK *expression. We also found a differential expression of *PRV1 *according to JAK2 V617F status and a correlation between *PRV1 *expression and platelet count in ET and PMF patients, as well as splenomegaly. Thus, our results suggest a link between *PRV1 *and intrinsic apoptosis pathway regulation and are in good agreement with previous reports about the association of the JAK2 V617F mutation with deregulation of apoptosis and disease phenotype [25].

**Figure 8:**
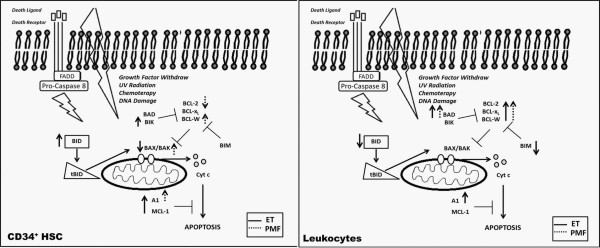
**Overview of the BCL2 family gene expression results**: (A) in CD34^+ ^HSC and (B) leukocytes from patients with ET (continuous arrows) and PMF (stippled arrows).

An illustrative overview of the gene expression results in CD34^+ ^HSC and leukocytes from ET and PMF is shown in Figure 8. This figure highlights the complexity of the apoptosis network in MPN patients. The analysis of the interaction of genes involved in the apoptotic machinery described here implies that apoptosis is deregulated and impaired in MPN patients since the majority of anti-apoptotic genes assessed are overexpressed, while concomitantly, some pro-apoptotic genes appear to be downregulated.

These observations are in line with previous results described by our group in Tognon et al. (2011) [24], which demonstrated that mononuclear cells from MPN patients are resistant to apoptosis, considering that patients' cells stimulated by numerous apoptotic inducers, such as actinomycin and cytarabin, showed less Annexin-V FITC staining compared to controls [24]. Therefore, deregulation of the intrinsic apoptosis pathway might contribute to ET and PMF physiopathology and myeloaccumulation.

MPN do not yet have a curative therapy so it is particularly relevant to consider the possibility of designing new drugs targeting apoptosis pathway. In this context, Zivny et al. (2010) reviewed this subject emphasizing the importance of developing new and more effective target cancer therapies with the potential of inhibiting the anti-apoptotic BCL2 family members or enhancing pro-apoptotic proteins expression [20]. These approaches might impair evasion of tumor cell to apoptosis processes or might sensitize cells to apoptosis. Taken together our results suggest that ET and PMF treatment could not be restrict to JAK2 inhibitors, considering that there are other molecular mechanisms involved in MPN pathogenesis, in addition to JAK2 mutation. Maybe in the future, JAK2 inhibitor treatment must be associated with new target therapies, such as anti-apoptotic BCL2 family members' inhibitors or pro-apoptotic enhancers, for better patients ' response to therapy.

## Conclusion

CD34^+ ^HSC and leukocytes from ET and PMF patients displayed a deregulation in expression levels of BCL2 family members, which are correlated with JAK2 V617F mutation and *PRV1 *mRNA levels. Our findings suggest that these alterations may contribute to increased resistance to apoptosis and to myeloaccumulation in ET and PMF patients.

## Competing interests

The authors declare that they have no competing interests.

## Authors' contributions

RT designed and performed experiments, analyzed data and wrote the paper. EPLG, RPN, NSN, AFF and PVBP performed some of the cell isolation, RNA extraction and gene expression assays. MAZ, EXS, BPS and MS selected the patients included in this study and collected the bone marrow samples for CD34^+ ^cell isolation. NSN, MAZ, DTC, AMS and SK performed real-time experiments, discussed the results and revised the paper. FAC conceived the project, created the study design, sought funding and wrote the paper. All authors critically reviewed the manuscript.
